# The persistent proatlantal artery: types and subtypes

**DOI:** 10.1007/s00276-026-03880-4

**Published:** 2026-04-06

**Authors:** Mugurel Constantin Rusu, Răzvan Costin Tudose, Alexandra Diana Vrapciu

**Affiliations:** 1 Division of Anatomy, Department 1, Faculty of Dentistry, “Carol Davila” University of Medicine and Pharmacy, Bucharest, 050474 Romania; 2 Clinic of Ophthalmology, University Emergency Hospital, Bucharest, 020021 Romania

**Keywords:** Persistent proatlantal artery, Carotid-vertebrobasilar anastomosis, Vertebral artery hypoplasia, External carotid artery, Computed tomography angiography, Vascular classification, Embryology

## Abstract

**Background:**

The persistent proatlantal artery (PPA) is the most caudal of the four persistent carotid–vertebrobasilar anastomoses and is frequently misclassified because “Type I/Type II” terminology variably reflects arterial origin and/or cervical course.

**Objective:**

To synthesise embryology, imaging criteria, and clinically relevant PPA variants, and to harmonise nomenclature including Cohen’s Type II “spinal” and “occipital” subtypes.

**Methods:**

A Scale for the Assessment of Narrative Review Articles (SANRA)-guided narrative review of PubMed/MEDLINE and Google Scholar (inception to February 2025) was performed. Data extracted included origin (internal carotid artery, external carotid artery [ECA], or common carotid artery), relationship to the C1 transverse foramen, vertebral artery (VA) status, occipital artery (OA) incorporation, and procedural relevance. An illustrative computed tomography angiography (CTA) index case was included.

**Results:**

Evidence is dominated by case reports and small series. PPA is very uncommon (reported incidence ~ 0.01% in multi-slice CTA cohorts) and often accompanies ipsilateral VA hypoplasia/aplasia, sometimes providing the dominant posterior fossa supply. Course-forward interpretation distinguishes canonical Type I suboccipital pathways from Type II ECA/occipital-system pathways, including “occipital” Type II channels that bypass transverse foramina and can mimic Type I. In the index case, a 73-year-old woman had an ECA-origin Type II (occipital subtype) PPA that gave rise to the OA and continued as the extracranial VA before entering the skull via the foramen magnum.

**Conclusion:**

Standardised, course-based reporting, explicitly documenting origin, transverse foramen traversal, and posterior circulation dependence, reduces diagnostic ambiguity and supports safer planning for ECA interventions, carotid endarterectomy, and craniovertebral junction/skull-base surgery.

## Introduction

During embryonic development, four transient anastomotic vessels connect the primitive carotid arteries to the longitudinal neural arteries: the primitive trigeminal, otic, hypoglossal, and proatlantal intersegmental arteries, arranged from superior to inferior [22, 32, 67]. Except for the extracranial proatlantal channel, these embryonic carotid-vertebrobasilar connections are typically named according to their relationship to adjacent cranial nerves [48]. These vessels are essential for establishing blood flow to the developing vertebrobasilar arterial system. Persistence of these embryonic arteries into adulthood results in persistent carotid-basilar anastomoses [30, 58, 63] (Table 1).

The internal carotid artery (ICA), external carotid artery (ECA) and, more rarely, common carotid artery (CCA) may give rise to a persistent proatlantal artery (PPA), a remnant of the embryonic carotid-vertebrobasilar anastomoses that transiently connect the carotid circulation with the developing vertebrobasilar system during early hindbrain vascularisation [58, 63]. In normal development, these fetal channels regress within a short developmental window as the vertebral arteries (VAs) mature. Systematic evaluation of the craniocervical circulation has highlighted that suboccipital (V3) and intradural (V4) VA variants are not uncommon on contemporary cross-sectional angiography, and that the differential for unusual posterior fossa inflow can include rare persistent embryological anastomoses, such as the PPA [56].

In this narrative review, we synthesise embryological concepts, imaging definitions, and clinically relevant variants of persistent proatlantal arteries (PPAs), with emphasis on harmonising the ‘Type I’ versus ‘Type II’ nomenclature and recognising hybrid occipital-system configurations. We also include an illustrative index case demonstrating a Type II PPA (occipital subtype) giving rise to the occipital artery (OA), and we highlight practical classification and procedural-planning pitfalls [9, 14, 58, 63].

**Table 1:** Comparison of major persistent carotid-vertebrobasilar anastomoses

Vessel/type	Role & prevalence	Typical course / key features	Clinical / imaging notes	Citations
Primitive trigeminal artery (PTA)	Most common persistent carotid–basilar anastomosis; ~0.1–0.6% in angiographic series [32, 35, 59]	Connects the intracavernous ICA to the basilar artery; often with hypoplastic basilar caudal to junction and hypoplastic/absent ipsilateral PCoA [17, 32, 59,66]	Often incidental; associated with aneurysms, AVMs, carotid–cavernous fistula, stroke; important for skull-base and endovascular planning [17, 24,32,59]	[17, 23, 32, 35,59, 61, 66]
Persistent trigeminal artery variant (PTAV)	A variant in which ICA supplies cerebellar artery directly (e.g., AICA, SCA) without an intervening basilar segment; prevalence up to ~ 0.2% [32, 35]	Arises from the cavernous ICA and terminates in a cerebellar artery, bypassing the basilar; it can be unilateral or very rarely bilateral [35]	May cause or contribute to neurovascular conflict, aneurysms; an important route for endovascular access to the posterior fossa [32, 35, 59]	[32, 35, 59]
Persistent hypoglossal artery (PHA/PPHA)	Rare carotid–basilar anastomosis; estimated 0.02–0.26% [61, 62]	Large branch of cervical ICA (occasionally ECA/CCA) entering skull via hypoglossal canal to join basilar artery [61, 62]	Frequently associated with hypoplastic/aplastic VAs; increased risk of aneurysm, AVM, and ischemia if carotid flow is compromised [21, 52, 61, 62]	[21, 32, 51, 52, 61, 62]
Persistent otic (acoustic) artery	Earliest to regress and rarest in adults [32, 62]	From the petrous ICA to the basilar artery through the internal acoustic meatus (course parallels VII–VIII nerve complex) [32, 62]	Extremely rare; mainly of theoretical/embryologic interest but critical if present at skull-base surgery or embolisation [32, 62]	[23, 32, 62]
Proatlantal intersegmental artery (ProA)	Caudal carotid–vertebral anastomosis; persistence is rare, but its remnant forms part of the OA [32, 35, 53]	When persistent, it connects the carotid system to the VA in the upper cervical region; two types: Type I from ICA, Type II from ECA, both entering the skull via foramen magnum and joining V4 (type I) or V3 (type II) segments [35, 61]	Persistence is usually accompanied by hypoplastic VAs; important collateral to the posterior circulation and relevant in cervical/suboccipital and foramen-magnum surgery, and in vertebral or carotid occlusive disease [35, 53, 61]	[32, 35, 53, 61]

AICA, anterior inferior cerebellar artery; AVM, arteriovenous malformation; BA, basilar artery; CCA, common carotid artery; ECA, external carotid artery; ICA, internal carotid artery; OA, occipital artery; PCoA/PCoA(s), posterior communicating artery/arteries; PHA/PPHA, (persistent) hypoglossal artery; PTA, persistent (primitive) trigeminal artery; PTAV, persistent trigeminal artery variant; SCA, superior cerebellar artery; VA, vertebral artery

The PTA, the commonest persistent carotid-basilar anastomosis, can be stratified by haemodynamic supply. Five types were defined, based on which posterior circulation territories are supplied by the basilar branches and the posterior communicating arteries [65].

Olry and Haines (2004) reported that the PPA was first described anatomically by Gottschau in 1885 and that, on angiography in 1960, Luccarelli and de Ferrari identified a variant arising from the ECA. They further noted that this observation prompted Lie to distinguish two PPA types: those arising from the ICA and those arising from the ECA [39]. In an early angiographic and embryologic synthesis, Abe and Suzuki (1964) defined the PPA as arising from the ICA between the C3 and C5 levels, coursing above the atlas, and entering the skull via the foramen magnum, typically accompanied by hypoplastic or absent VAs [1].

Hutchinson and Miller (1970) subsequently described an angiographic case of a PPA arising from the cervical ICA. They reviewed the developmental anatomy, emphasising that this vessel is prone to misinterpretation as a persistent hypoglossal artery. They noted that, unlike the hypoglossal artery, the PPA forms the VA extracranially and enters the skull via the foramen magnum [25].

The PPA, also termed the persistent proatlantal intersegmental artery, is the most caudal subtype of these persistent anastomoses; the persistent otic (acoustic) artery is generally regarded as the rarest [7]. At the 4 mm embryonic stage, the PPA arises as an anterior branch of the dorsal aorta, forming an anastomosis between the primitive ICA and the bilateral longitudinal nerve arteries supplying the hindbrain [67]. Typically, the PPA regresses as the posterior communicating and VAs develop, and persistence results in a carotid-vertebrobasilar conduit that may partially substitute for a hypoplastic or absent VA [32].

In a retrospective multislice computed tomography angiography (MSCTA) cohort, the incidence of PPA was reported as 0.01% [67]. In their magnetic resonance (MR) angiography-based pictorial review, Luh et al. (1999) noted that approximately 40 cases had been reported in the literature. In a retrospective series of 600 cerebral angiograms, two PPAs (0.33%) were identified: one Type I and one Type II (0.16% each) [41]. When the PPA persists, the ipsilateral VA is consistently hypoplastic or absent, and the PPA can serve as the predominant posterior fossa supply in about 75% of cases [22, 67].

The present review follows the Lasjaunias numbering convention in which the proatlantal intersegmental artery corresponds to the first cervical segmental artery (arising between the occiput and C1), and the “C1 intersegmental artery” (Type II) corresponds to the first cervical intersegmental artery proper, consistent with the embryological framework of Padget (1948) and as adopted by Lasjaunias et al. (1978) [30, 42]. Readers should note that alternative numbering systems exist in the older literature, and that some apparent discrepancies between reports reflect different conventions for counting cervical segmental arteries rather than true morphological disagreements.

The different arterial origins of persistent proatlantal channels can be explained by the embryological relationships of the cervical segmental arteries [30, 32]. At the 4–12 mm embryonic stage, the proatlantal intersegmental artery arises from the dorsal aorta between the occiput and C1 and connects the primitive ICA to the longitudinal neural arteries [42, 67]. Normally, this channel regresses as the VAs consolidate from longitudinal anastomoses of the cervical intersegmental arteries [32, 35, 58]. ICA-origin persistence (Type I) results when the native proatlantal–ICA junction fails to regress [30, 63]. ECA-origin persistence (Type II) occurs when the developing external carotid/occipital system annexes the proatlantal channel, so that the persistent vessel originates from the ECA or its occipital branch rather than directly from the ICA [2, 14, 30]. CCA-origin persistence reflects retention at a level proximal to the definitive carotid bifurcation [19, 26, 32]. In each scenario, the distal course of the persistent channel is determined by the degree to which adjacent cervical segmental arteries have been incorporated or have regressed, accounting for the variable relationship to the C1 transverse foramen [14, 30, 42].

## Methods

This narrative review was conducted in accordance with the Scale for the Assessment of Narrative Review Articles (SANRA) guidelines [5]. PubMed/MEDLINE and Google Scholar were searched from inception through February 2025 using the terms *persistent proatlantal artery*, *persistent proatlantal intersegmental artery*, *persistent carotid-vertebrobasilar anastomosis*, *Type I/Type II proatlantal artery*, and *C1 intersegmental artery*, individually and in combination. Reference lists of key articles were hand-searched for additional records. No language restrictions were applied.

Articles were excluded if they did not provide sufficient anatomical detail to determine the vessel’s origin, cervical course, or relationship to the C1 transverse foramen. Conference abstracts without accompanying full-text data were also excluded. When the type label assigned by the original authors was inconsistent with the described morphology (e.g., an ECA/occipital-system origin PPA with a suboccipital course labelled “Type I”), the case was re-interpreted against the Lasjaunias angiographic classification (course-based) and the Cohen Type II subtype framework (“spinal” vs. “occipital”). In all tables, the original reported label is presented alongside the current morphological interpretation proposed by the present authors, so that the reader can distinguish between source data and reinterpretation.

Articles were included if they reported original case descriptions, imaging series, embryological analyses, or reviews about persistent proatlantal arteries or related carotid-vertebrobasilar anastomoses. From each report, PPA type, artery of origin, cervical course, relationship to the C1 transverse foramen, VA status, OA relationship, associated anomalies, and clinical or procedural relevance were extracted where available. Findings were tabulated (Tables 1, 2, 3 and 4) and synthesised narratively. Cases in which the reported label was inconsistent with the described morphology were re-interpreted against the Lasjaunias classification and the Cohen Type II subtype framework (spinal versus occipital). An illustrative index case of a Type II PPA (occipital subtype) identified on CTA was included as a didactic vignette. The available evidence consists predominantly of case reports and small retrospective series; conclusions should be interpreted accordingly. The illustrative index case was drawn from an ethically approved study (approval no. 10540/16.02.2022, University Emergency Hospital, Bucharest); all patient data were fully anonymised and no informed consent was required for the use of anonymised imaging data in this context.

### Results / literature synthesis

Two classification systems exist for PPA. In the angiographic classification of Lasjaunias, Type I and Type II are distinguished primarily by their cervical course: Type I follows a suboccipital horizontal sweep to enter the skull via the foramen magnum without traversing cervical transverse foramina, whereas Type II courses more laterally and typically incorporates the C1 transverse foramen before joining the V3 segment. Although Type I is often summarised as ICA-origin and Type II as ECA-origin, origin alone is insufficient for classification. Type I most commonly arises from the cervical ICA; rare ECA-origin Type I has been documented [3, 12, 27]. However, the ECA/occipital-system origin configuration labelled as ‘Type I’ in these reports (and in the ECA/occipital-origin access case of Nariai et al., 2021) is morphologically similar to the bilateral ECA-origin Type II phenotype described by Menon et al. (2013) and corresponds to Cohen’s Type II occipital subtype (Type II origin with Type I-like suboccipital course and no transverse foramen traversal) [14, 38, 44]. A Type I arising from the common carotid artery has also been demonstrated on CTA/angiography in association with aberrant right subclavian artery and VA aplasia [26], supporting earlier inclusion of CCA/bifurcation origin among proposed diagnostic criteria in pictorial reviews [32] and later reviews [40, 63]. Vasovic et al. (2009) cite both Luh et al. (1999) and Anderson and Sondheimer (1976) in support of an ECA-origin Type I; however, Luh et al. (1999) provide diagnostic criteria in a review context (including a persistent hypoglossal artery) rather than a primary ECA-origin Type I case [3, 32, 63]. Accordingly, the reports of Anderson and Sondheimer (1976), Choi, et al. (2018) and Ito et al. (2023) represent the principal case-based evidence of an ECA/occipital-system origin PPA with a Type I-like course [3, 12, 27]. More recently was proposed a novel classification based on posterior circulation blood supply, categorising PPA into four types according to the relative contributions of the persistent artery, VA, and posterior communicating artery [67].

Some authors did not explicitly specify whether the persistent proatlantal channel corresponded to Type I or Type II, particularly in earlier angiographic and embryologic reports [1, 3, 15, 18, 33, 54, 57].

Beyond the classical dichotomy, contemporary reports emphasise that persistent proatlantal channels can present with atypical origins, hybrid courses, or bilateral or asymmetric persistence (Table 2). Cohen et al. (2011) refined Type II morphology by distinguishing a ‘spinal’ subtype that ascends via one or more cervical transverse foramina (typically incorporating the C1 transverse foramen) from an ‘occipital’ subtype that bypasses transverse foramina and directly reaches the VA groove of C1, thereby mimicking the Type I suboccipital sweep [14]. This ‘occipital’ Type II configuration has also been labelled ‘mixed’ (Type II origin with Type I-like course). It is exemplified by the cases reported by Ma et al. (2019) and Boukobza and Laissy (2025), in which an external carotid-origin PPA courses over the posterior arch of C1 without traversing any transverse foramen before entering the skull via the foramen magnum [9, 14, 34]. Menon et al. (2013) described bilateral Type II persistent proatlantal intersegmental arteries arising from the external carotid arteries, with the occipital arteries arising from the persistent trunks at the suboccipital turn, where the vessels course medially to form the V3/V4 segments [44]. Several reports labelled as ECA-origin ‘Type I’ PPAs [12, 27, 38] share this occipital-channel morphology and are best interpreted as Cohen Type II (occipital subtype) rather than strict Lasjaunias Type I variants. In practice, Type II PPA may also be reported as a VA of external carotid origin [11, 18], and rare common carotid origins have been documented for Type II channels [19]. Type II channels may enter cervical transverse foramina below C1 (e.g., C3) before ascending to C1 [4]. Kurose et al. (1990) provided a classic example of the ‘spinal’ Type II phenotype, with an external carotid origin channel passing through the C1 transverse foramen before joining the VA. Conversely, Uchino and Tokushige (2022) reported a relatively small Type II PPA coexisting with a normally developed ipsilateral VA, underscoring that VA aplasia/hypoplasia is frequent but not obligatory [60]. A Type I-like PPA arising from a common trunk of the OA from the ECA was described [40]. It was used as an access route for distal PICA aneurysm treatment, illustrating that OA incorporation can confound simplified ‘origin-based’ labels [40]. Bilateral or concurrent Type I and Type II PPAs have also been reported, often with the OA arising from the persistent channel(s) [16, 45, 46]. Bilateral persistence of proatlantal arteries with absence of both VAs has also been reported [33].

Our index case further illustrates this ‘occipital’ Type II phenotype: an ECA-origin PPA bypassed the C1 transverse foramen, gave rise to the ipsilateral OA, and continued as the V3 vertebral segment before entering the skull via the foramen magnum, supporting that transverse foramen traversal is not mandatory for Type II classification [3, 14, 67].

**Fig. 1:**
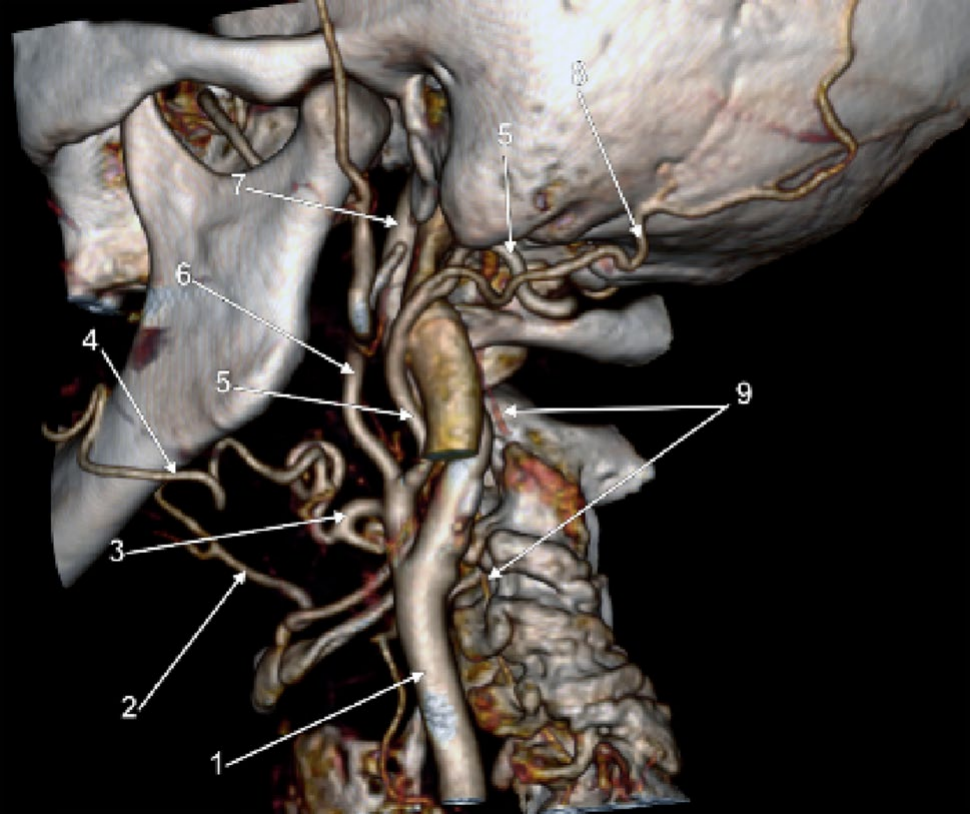
Left persisting proatlantal artery with external carotid origin and direct course to the atlantooccipital space. Three-dimensional volume rendering. Left postero-infero-lateral view. (1) common carotid artery; (2) lingual artery; (3) linguofacial trunk; (4) facial artery; (5) persisting proatlantal artery; (6) external carotid artery; (7) internal carotid artery; (8) occipital artery; (9) V2 segment of the vertebral artery

*Index case (illustrative vignette)*: A 73-year-old woman underwent computed tomography angiography (CTA), in whom a left-sided Type II PPA (occipital subtype) was identified. The persistent channel originated from the posterior aspect of the left ECA opposite the C2/C3 intervertebral disc and ascended directly to the atlanto-occipital region without traversing the C1 transverse foramen (Fig. 1). In the upper neck, it gave off the left OA and then continued as the V3 segment of theVA, entered the dura through the foramen magnum, supplied the left posterior inferior cerebellar artery, and finally joined the dominant right VA to form the basilar artery. The subclavian-origin left VA was markedly hypoplastic along its cervical course, emphasising the potential for carotid-derived channels to contribute materially to posterior circulation haemodynamics in such variants.

**Fig. 2:**
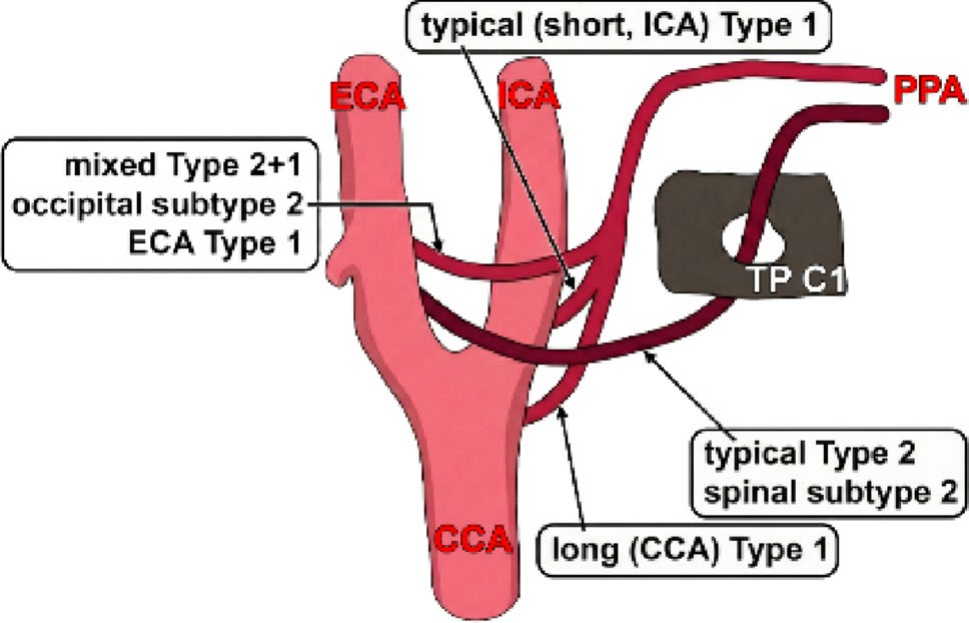
Harmonised types of the persisting proatlantal artery. CCA: common carotid artery; ICA: internal carotid artery; ECA: external carotid artery; PPA: persisting proatlantal artery; TP C1: transverse process of the atlas

**Fig. 3:**
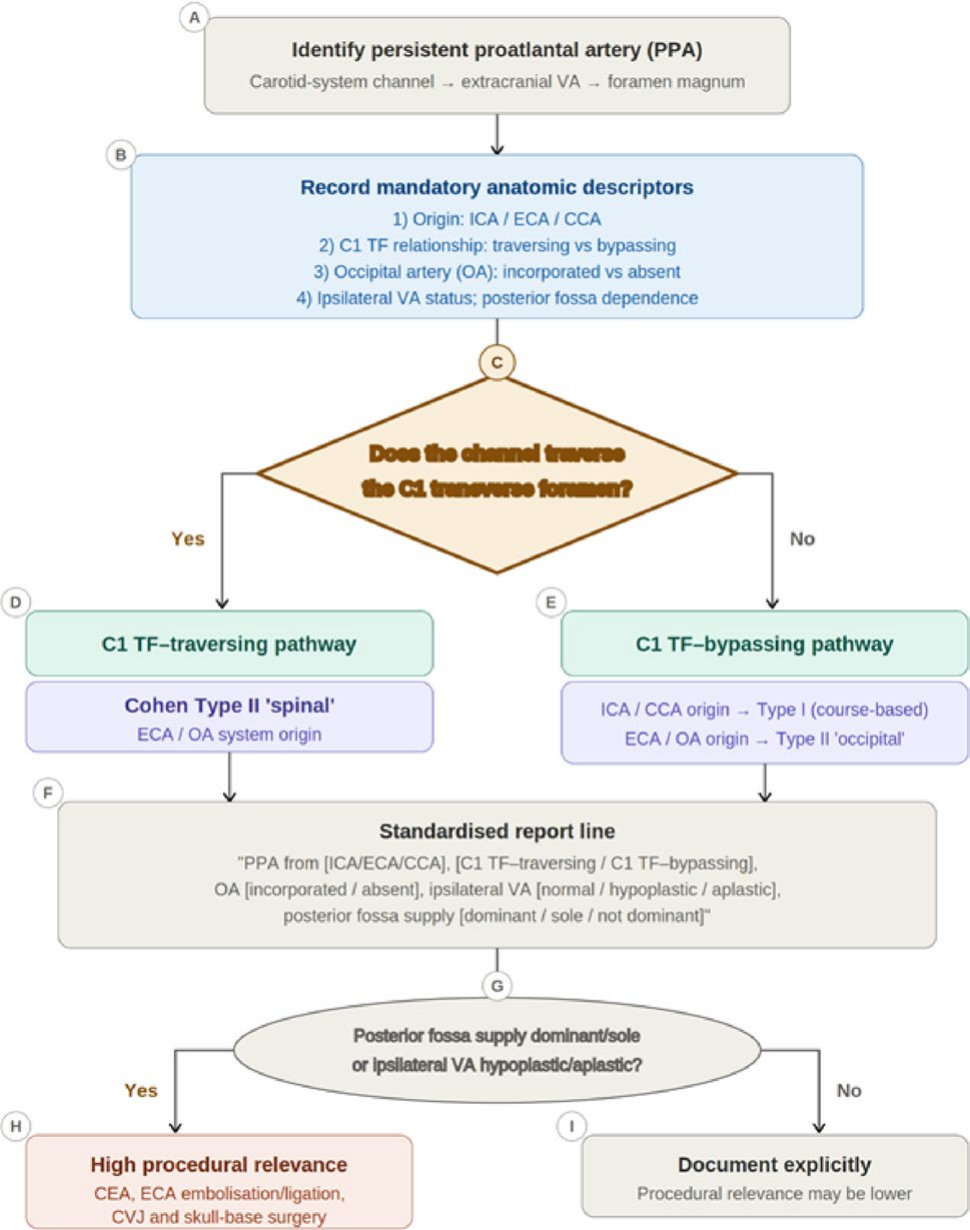
Persistent proatlantal artery (PPA) remains underrecognised because the traditional Type I/Type II dichotomy is inconsistently applied when based on origin alone. A course-based approach using the relationship to the C1 transverse foramen (C1 TF) more reliably separates C1 TF–traversing from C1 TF–bypassing pathways and accommodates Cohen’s Type II “spinal” and “occipital” subtypes. Standardised reporting of origin, C1 TF relationship, occipital artery (OA) incorporation, and ipsilateral vertebral artery (VA) status reduces nomenclature overlap and clarifies anatomic risk in carotid endarterectomy (CEA), external carotid artery (ECA) procedures, and craniovertebral junction (CVJ)/skull-base surgery

Figure 2 schematises a two-axis harmonisation of persistent proatlantal artery (PPA) nomenclature by separating the cervical course (Type I-like vs. Type II-like) from the carotid-system origin (ICA/ECA/CCA). The upper channel represents the canonical “short, ICA-Type I” configuration, in which the persistent channel arises from the cervical ICA and reaches the atlanto-occipital region with a Type I-like suboccipital sweep (i.e., without obligatory transverse foramen traversal). A second “long, CCA-Type I” pathway is shown to accommodate reported Type I-like courses with a more proximal carotid origin. By contrast, the right-sided pathway depicts the typical Type II (“spinal” subtype), in which an ECA/occipital-system artery ascends via the C1 transverse foramen (TP C1) before joining the V3 segment [14, 29]. Finally, the left-sided “mixed Type 2 + 1 / occipital subtype” channel highlights the key source of inconsistent labels in the literature: several cases described as ECA-origin “Type I” based on course [12, 27, 38] are morphologically congruent with the ECA-origin Type II phenotype described by Menon et al. (2013) and align best with Cohen’s Type II “occipital” subtype (a Type II origin with a Type I-like suboccipital course and no transverse foramen traversal), as also exemplified in “mixed” configurations reported by Ma et al. (2019) and Boukobza and Laissy (2025) [9, 14, 34, 44]. A standardised, course-based reporting algorithm derived from this two-axis classification is presented in Fig. 3.

**Table 2:** Anatomical comparison of PPA Type I and Type II (Lasjaunias’ classification)

Characteristic	Type I (Proatlantal intersegmental artery)	Type II (C1 intersegmental artery)
Synonyms	Proatlantal intersegmental artery; Persistent proatlantal intersegmental artery [55]	C1 intersegmental artery; Persistent first cervical intersegmental artery [29]
Embryological origin	Proatlantal intersegmental artery (first cervical segmental artery) [30]	First cervical intersegmental artery / proatlantal system [2, 32]
Artery of origin	Internal carotid artery (most common). ECA/occipital-system origin with a Type I-like suboccipital course has been reported [3, 12, 27, 38] and is best regarded as Cohen Type II (occipital subtype) rather than a distinct Type I entity [14, 44]. Rare origin from the common carotid artery has also been documented [26]; reviews include CCA/bifurcation among proposed diagnostic criteria [32, 63].	External carotid artery (proximal portion) [8, 30, 44, 60]
Level of origin	C2–C4 vertebral level, typically C2–C3 [7,32,67]. ECA-origin Type I at the C2–C3 disc has been demonstrated on CTA [12]. A CCA-origin Type I has been reported in association with an aberrant right subclavian artery [26].	Usually at C2–C3, but can arise at the C3–C4 disc level [2, 29, 46, 67]
Course	Ascends anteriorly along vertebral bodies to the occipitoatlantal space, then courses dorsally with a characteristic horizontal sweep [6, 22, 57]	Runs posteriorly, similar to the occipital artery course; crosses C1 or C2 obliquely; has a more tortuous path than Type I [8, 30, 60, 67]
Transverse foramen	Does NOT pass through the transverse foramen of any cervical vertebra [22, 27, 41, 57, 67]	Typically passes through the C1 transverse foramen before joining V3 (Cohen et al., 2011; ‘spinal’ Type II variant), but entry into lower transverse foramina (e.g., C3) and variable suboccipital courses are reported [2, 4, 8, 41, 67]. Cohen et al. (2011) also described an ‘occipital’ Type II subtype that bypasses transverse foramina and reaches the C1 vertebral groove (see Hybrid/mixed patterns).
Junction with VA	Joins the V4 segment (horizontal portion) of the VA in the occipitoatlantal space [55]	Joins the V3 segment of the VA under the C1 vertebra [8, 29]
Entry to skull	Foramen magnum [22]	Foramen magnum [8, 67]
Relative frequency	18.2% of PPA cases in the MSCTA series [67]. In a literature-based synthesis, Type I accounted for ~ 38% of reported PPA [63]. Review-based sources also quote a small fraction of PPA arising from the common carotid artery [32, 63], but a primary Type I case report with CCA origin was not identified in the sources included here. In a 600-angiography series, the incidence of Type I was 0.16% [41].	81.8% of PPA cases in the MSCTA series [67]. In a literature-based synthesis, Type II accounted for ~ 57% of reported PPA [63]. In a 600-angiography series, the incidence of Type II was 0.16% [41].
Associated VA status	Ipsilateral VA is absent in 100% of cases; contralateral VA is hypoplastic in ~ 27% [67]. Case reports demonstrate that bilateral VA absence can occur in Type I PPA [36, 57], and proximal VA aplasia may accompany CCA-origin Type I [26].	Ipsilateral VA is usually aplastic proximal to the anastomosis; contralateral VA is often hypoplastic, may terminate in PICA [2,29]; however, a relatively small Type II PPA with a normally developed ipsilateral VA has been described [60].
Key differentiating feature vs. hypoglossal artery	Horizontal suboccipital course with dorsal sweep; forms the VA extracranially and enters via foramen magnum. Origin is typically opposite C2/C2 interspace, lower than persistent hypoglossal artery (often opposite C1 or the C1/2 interspace), which enters the skull via the anterior condyloid (hypoglossal) canal [22, 25, 67].	Passage through C1 transverse foramen when present; visible on open-mouth anteroposterior (AP) view [29]
Hybrid/mixed patterns	Not part of the original Type I definition.	Hybrid/mixed (Cohen et al., 2011; ‘occipital’ Type II subtype): external carotid origin (Type II) but Type I-like suboccipital course over the posterior arch of C1 and skull entry via the foramen magnum without transverse foramen traversal; may give rise to an occipital branch/OA [9,14,34,44]. This phenotype also encompasses ECA/occipital-origin cases reported as Type I [12, 27, 38].

BA, basilar artery; C1–C4, cervical vertebral levels 1–4; CCA, common carotid artery; DSA, digital subtraction angiography; ECA, external carotid artery; FM, foramen magnum; ICA, internal carotid artery; OA, occipital artery; PPA/PPAs, persistent proatlantal artery/arteries; VA, vertebral artery; V1–V4, vertebral artery segments 1–4; V3/V4, vertebral artery segments 3/4

**Table 3:** Xiao et al. (2024) [67] proposed a classification based on posterior circulation blood supply

Type	Description	Vessel status	Frequency
Type 1	Persistent artery dominant; PPA is the primary supplier to the posterior circulation	Well-developed PPA and BA; VA variable; PCoA optional	36.4% (4/11 cases)
Type 2	Balanced type; posterior circulation supported by both PPA and contralateral VA	Normal PPA and contralateral VA; ipsilateral VA dysplastic; PCoA optional	36.4% (4/11 cases)
Type 3	Vertebral artery dominant; the contralateral VA mainly sustains the posterior circulation	Normal contralateral VA and BA; dysplastic ipsilateral VA and PPA; PCoA optional	9.1% (1/11 cases)
Type 4	PCoA blood supply type: posterior circulation predominantly via the posterior communicating artery	Dysplastic VA, BA, and PPA; well-developed bilateral PCoA	18.2% (2/11 cases)

BA, basilar artery; PCoA, posterior communicating artery; PPA, persistent proatlantal artery; VA, vertebral artery, Adapted from Xiao et al. (2024).

**Table 4:** Relationship of the occipital artery with proatlantal artery types

Characteristic	Type I (Proatlantal intersegmental artery)	Type II (C1 Intersegmental artery)
Embryological relationship	OA horizontal and distal portions are derived from the proatlantal artery. Akay et al. (2005) similarly noted that the horizontal segment of the VA and the horizontal/distal OA are derived from the proatlantal system [2]. When PPA Type I persists, it represents the rudimentary channel that should have become the proximal OA ([30, 55]. OA arising from the cervical ICA has been interpreted as a persistent proatlantal intersegmental artery [26, 54].	Type II PPA takes a similar course to OA. The peripheral branch of OA arises from the distal segment just proximal to the foramen magnum [60].
OA arising from PPA	YES – documented. Tanaka et al. (1983): the OA arose at/near the PPA–VA junction (proximal horizontal VA segment) in a Type I PPA arising from the ICA [55].	YES – documented. Uchino et al. (2022): OA arises from the distal segment of Type II PPA, just proximal to FM [60].
Mechanism	Shared developmental substrate with the occipital system: Luh et al. (1999) and Akay et al. (2005) note that the horizontal and distal OA segments are derived from the proatlantal intersegmental artery, supporting how persistence of the proatlantal channel can incorporate OA segments into a carotid-vertebrobasilar conduit [2, 30, 32]. Anderson and Sondheimer (1976) demonstrated an occipital branch arising from an ECA-origin Type I PPA, consistent with recruitment of the occipital system into the persistent channel [3].	If the ECA-derived segment persists as Type II PPA and connects to VA, the distal OA branches off the persistent vessel [30, 32].
Case details	Tanaka et al. (1983): 69-year-old woman with coexisting Type I PPA (from ICA at C2) and OA arising at/near the PPA–VA junction; contralateral persistent trigeminal artery with aneurysm at its ICA origin; hypoplastic vertebral/basilar system supplied segmentally by the persistent channels [55].	Uchino et al. (2022): 81-year-old man with Type II left PA; OA arose from the proximal segment and fused with VA at the FM level. Normally developed ipsilateral VA (unusual) [60].
Additional reports/variants	An OA arising from the persistent channel has been described in combined configurations, including a classic ECA-origin PPA with an occipital branch arising from the anomalous vessel [3], concurrent Type I (ICA origin) and Type II (ECA origin) PPAs with occipital arteries arising from the anomalous vessels bilaterally [46], and bilateral proatlantal persistence arising from OA trunks with absence of both VAs [33]. Several ECA/occipital-origin channels reported as ‘Type I’ [12, 27, 38] match Cohen Type II (occipital subtype) and therefore further support an occipital-system basis for apparent Type I/Type II overlap [14].	Hybrid and Type II-related presentations further illustrate the OA–PPA relationship: bilateral Type II persistent proatlantal intersegmental arteries with occipital arteries arising from the persistent trunks [44]; a mixed PPA (Type II origin with Type I course) giving rise to the OA [9]; Type II (occipital subtype) with an occipital musculocutaneous branch arising over C1 and an upper cervical course that bypassed transverse foramina [14]; and Type II PPA of ECA origin associated with vein of Galen malformation where the OA arose from the PPA [45]. In Capel et al. (2013), an ECA-origin of the VA with a remnant Type II PPA gave off the ipsilateral OA, which supplied a Cognard type IV dural arteriovenous fistula [11].

FM, foramen magnum; ICA, internal carotid artery; OA, occipital artery; PPA, persistent proatlantal artery; VA, vertebral artery

Lasjaunias, Théron and Moret (1978) explicitly linked the OA anatomy to the proatlantal system, proposing that the proximal ascending OA (including the muscular pedicle of the second intervertebral space) corresponds to the second segmental artery [30]. In contrast, the horizontal and distal ascending occipital segments reflect persistence of the first segmental artery. In this framework, the PPAs (Types I and II) represent maximal persistence of these embryonic channels, while the more common occipitovertebral anastomoses represent minimal persistence [30]. Suzuki et al. (1979) similarly interpreted an anomalous OA arising from the cervical ICA as a PPA, supporting an embryological continuity between the occipital system and the proatlantal channel [54]. Lui et al. (1987) reported bilateral proatlantal persistence arising from the proximal OAs, with absence of both vertebral arteries, underscoring the surgical relevance of recognising occipitovertebral recruitment [33].

**Table 5:** Selected reports highlighting PPA-related arterial origins and hybrid patterns

Study	PPA/variant	Key arterial origins/associations	Clinical relevance
Abe and Suzuki [1]	Type not specified (early angiographic definition of PPA)	Early angiographic/embryologic description defining persistent proatlantal artery as an ICA-derived channel arising between C3 and C5, coursing above the atlas, and entering the skull through the foramen magnum, with VAs frequently hypoplastic or absent.	Provides foundational diagnostic criteria (origin level and foramen magnum entry) and the association with the VA hypoplasia/aplasia, supporting modern differentiation from persistent hypoglossal artery.
Conforti et al. [15]	Type not specified (primitive cervical segmental artery; PPA mimic)	Anomalous carotid–vertebral anastomosis interpreted as a primitive cervical segmental artery; branch from ICA at C2 level with a double-curved extracranial course joining the VA at C1; normal hypoglossal canal; hypoplastic ipsilateral transverse process of the atlas.	Classic radiological differential diagnosis against persistent hypoglossal artery, relevant to contemporary CTA/DSA interpretation and to procedural risk during ICA ligation/occlusion when the vertebrobasilar perfusion depends on the anomalous channel.
Flynn [18]	ECA-origin dominant VA (historical description)	The dominant VA is described as arising from the ECA (interpretable as a persistent proatlantal channel).	Highlights variability in angiographic terminology and its relevance to surgical ligation planning.
Hutchinson and Miller [25]	Type I PPA (ICA origin; angiographic case)	Cervical ICA bifurcated giving rise to a large posterior branch (reported at upper cervical level), forming the extracranial VA above C1; entered the skull via the foramen magnum and filled the basilar artery, with no contribution from the contralateral VA demonstrated. The authors emphasised angiographic differentiation from a persistent hypoglossal artery based on foramen magnum entry and a typical lower cervical origin level.	Classic description and differentiation criteria; underscores potential posterior circulation dependence on carotid inflow and the need for accurate classification during angiography and neck surgery.
Anderson and Sondheimer (1976) [3]	ECA-origin PPA with Type I-like suboccipital course (Cohen Type II ‘occipital’ subtype / mixed configuration)	Arises from the external carotid artery at the inferior margin of C2; it supplies the vertebrobasilar system, gives rise to an occipital branch, and enters the skull via the foramen magnum without traversing cervical transverse foramina.	Seminal angiographic description emphasising differentiation from persistent hypoglossal artery; highlights procedural risk of ECA ligation/embolisation and carotid surgery when posterior circulation depends on the persistent channel.
Suzuki et al. [54]	Type not specified (persistent proatlantal intersegmental artery; OA-from-ICA variant)	Case 1: a common trunk bifurcated into persistent hypoglossal and proatlantal intersegmental arteries, with the hypoglossal branch entering via the hypoglossal canal and the proatlantal branch running dorsolaterally with an occipital-type course. Case 2: an anomalous OA arising from the cervical ICA was interpreted by the authors as a persistent proatlantal intersegmental artery, based on its identical course and distribution. It was associated with an intracranial aneurysm and a frontal AVM.	Emphasises diagnostic differentiation among carotid–vertebrobasilar anastomoses and supports the concept that an ‘OA from the ICA’ may represent a proatlantal remnant relevant to embolisation/surgical planning.
Tsukamoto et al. [57]	Type I PPA (ICA origin) with bilateral VA absence	Right ICA-origin proatlantal intersegmental artery; horizontal suboccipital course over atlas without transverse foramen traversal; enters foramen magnum; both VAs absent; detected during evaluation of ruptured ACom aneurysm.	Demonstrates extreme posterior circulation dependence on carotid inflow; compromise of ICA (e.g., stenosis/CEA) may endanger vertebrobasilar perfusion.
Tanaka et al. [55]	Type I PPA with OA association and coexisting persistent trigeminal artery aneurysm	Left ICA-origin PPA at C2 joins the horizontal VA segment without passing through transverse foramina; the OA arises at/near the PPA–VA junction; coexisting persistent trigeminal artery with aneurysm at its ICA origin.	Illustrates coexistence of multiple persistent fetal anastomoses and aneurysmal disease; supports embryologic continuity between the proatlantal system and the OA.
Lui et al. [33]	Type not specified (bilateral ECA/occipital-origin proatlantal arteries; absent VAs)	Bilateral anomalous vessels arose from the proximal occipital arteries (ECA system). They joined the horizontal portion of the VA, with a common occipital trunk and absence of both VAs on aortic arch angiography. The authors highlight differentiation from the hypoglossal artery (foramen magnum entry with dorsal loop) and from the first cervical intersegmental artery (more lateral ascent on AP view). They noted that the proximal OA might be derived from the proatlantal system.	Illustrates the extreme dependence of the posterior circulation on bilateral occipital/proatlantal channels and reinforces the importance of recognising such anastomoses before ECA ligation or embolisation.
Kurose et al. [29]	Type II PPA (‘spinal’ subtype)	Large ECA-origin channel joins VA under C1 and passes through the C1 transverse foramen; ipsilateral VA aplasia and contralateral VA hypoplasia (terminating in PICA).	Reported with ruptured MCA aneurysm and haemorrhage; underscores the need to distinguish Type II (C1 transverse foramen) from Type I-like ‘occipital’ variants when planning cervical/craniovertebral interventions.
Kolbinger et al. [28]	Type I with carotid occlusive disease	Right Type I proatlantal artery arose from the right ICA at C2 and supplied vertebrobasilar circulation in the setting of right ICA occlusion proximal to the PPA and 70% left ICA stenosis; no VAs were demonstrated, and both PCoAs were aplastic, with retrograde cross-filling via the anterior communicating artery.	Illustrates haemodynamic dependence on carotid inflow and operative relevance: thrombendarterectomy was performed without interrupting retrograde flow via the PPA, with neurological recovery.
Luh et al. [32]	Review / diagnostic criteria (MR angiography)	Lists PPA diagnostic criteria: origin from CCA bifurcation, ECA or ICA at C2–C4; joins VA suboccipitally; traverses foramen magnum; notes shared developmental relationship with OA segments.	Supports origin-level spectrum (CCA/ICA/ECA) as imaging diagnostic criteria and highlights embryologic links to the OA, but does not itself constitute a primary case report for Type I CCA origin; the CCA/bifurcation origin is repeated in later reviews (e.g., Vasovic et al., 2009).
Basekim et al. [7]	Type I PPA with bilateral absence of the external carotid arteries	Right ICA-origin Type I PPA at C2 with foramen magnum entry; right VA aplasia and left VA hypoplasia; external carotid arteries absent bilaterally with ECA branches arising from the common carotid arteries.	Rare combined cervical/arch anomalies that can affect surgical planning; underscores that carotid–vertebrobasilar channels may coexist with major extracranial arterial aplasia.
Gumus et al. [22]	Bilateral Type I PPA with VA absence	Bilateral ICA-origin Type I PPAs at C2; both VAs are absent at their origins; persistent channels form the horizontal vertebral segments and enter the skull via the foramen magnum.	Highlights haemodynamic relevance (possible embolic source) and reinforces key differentiators from persistent hypoglossal artery (suboccipital horizontal course and foramen magnum entry).
Akay et al. [2]	Type II (ECA origin) on MSCTA	Type II PPA arising from ECA around C3–C4 level; traversed C1 transverse foramen and continued as ipsilateral VA; proximal ipsilateral VA segments not visualised on Doppler.	Demonstrates non-invasive computed tomography angiography (CTA) characterisation and emphasises relevance for ischaemic presentations and planning of endarterectomy/angioplasty.
Purkayastha et al. [45]	Type II (ECA origin) with venous malformation	Type II PPA(s) associated with vein of Galen malformation; OA arose from PPA in a unilateral case.	Reinforces association with complex vascular malformations and altered haemodynamics.
Li et al. [31]	Type II PPA (ECA origin, continued as VA)	Posterior circulation supplied by a vessel from the right ECA shortly after CCA origin; bilateral proximal (V1–V2) VA segments are absent; no left VA; objective pulsatile tinnitus and cervical bruit.	Neuro-otologic presentation indicates haemodynamic dependence and supports delineation before carotid endarterectomy/stenting, or neck manipulation.
Arraez-Aybar et al. [4]	Type not specified (angiographic description; retrospectively consistent with ECA-origin Type I course)	Type II PPA arising from ECA cranial to facial artery (C3–C4); loop at C3 and entry into transverse foramina (C3–C1); associated with the posterior cerebral artery AVM.	Supports the variability of transverse foramen entry level and association with congenital vascular malformations
Cohen et al. [14]	Type II ‘occipital’ subtype (mixed Type II origin + Type I-like course)	Large anastomotic vessel arising from proximal ECA via OA, connecting to distal VA at C1; bypassed cervical transverse foramina and directly contacted the C1 vertebral groove; bilateral absence of cervical VAs; musculocutaneous occipital branch over C1. Proposed ‘occipital’ (bypasses transverse foramina; mixed course) versus ‘spinal’ (enters transverse foramina) Type II variants.	Demonstrates that some Type II channels can mimic Type I course and supports recognising occipital-branch incorporation in posterior circulation–dependent patients.
Capel et al. [11]	Type II remnant (ECA-origin VA)	Vertebral artery originating from ECA; ipsilateral OA originated from VA and supplied a Cognard type IV dural arteriovenous fistula.	Demonstrates haemorrhagic presentation and needs to recognise OA/VA variants.
Menon et al. [44]	Type II (ECA origin; OA from persistent trunk)	Bilateral Type II PPAs arising from the ECAs just above their origins; both vessels ascended and then turned medially at the suboccipital level to form the V3/V4 segments; the occipital arteries originated from the persistent trunks at the juncture where they turned medially. The VAs were hypoplastic/absent, and the posterior circulation was supplied via persistent channels.	Demonstrates bilateral haemodynamic dependence on carotid-derived vertebrobasilar flow and the frequent incorporation of the occipital system into Type II anatomy, supporting Cohen’s occipital-subtype concept and helping explain ‘Type I’ labels applied to similar ECA/occipital-origin courses.
Montechiari et al. [36]	Type I with bilateral VA absence	Right ICA-origin Type I PPA with absence of both VAs; right fetal-type PCA and absence of left PCoA.	Demonstrates extreme posterior circulation dependence on the ipsilateral carotid–PPA axis; has implications for carotid disease management and perioperative risk during carotid procedures.
Saito et al. [50]	Type I PPA with aortic arch anomalies	Anastomotic artery between ICA and distal VA passing through foramen magnum without transverse foramina; associated aberrant right subclavian artery, hypoplastic ipsilateral VA, left VA arising directly from the aortic arch, and azygos ACA aneurysm.	Supports evaluation of the aortic arch and intracranial circulation when PPA is present, given the potential coexistence of vascular anomalies relevant to surgery and endovascular access.
Buljan et al. [10]	Type I with extracranial aneurysm and circle of Willis anomaly	Type I PPA (origin at C2–C3) provided the only posterior circulation supply; associated with a fusiform subclavian artery aneurysm, aplasia of the precommunicating ACA (A1) segment, and extensive carotid atherosclerosis/calcification.	Demonstrates clinically important coexisting anomalies and perioperative vulnerability; post-endarterectomy course was complicated by a large hemispheric infarction and death, supporting a cautious strategy in carotid surgery when PPA is dominant.
Choi et al. [12]	Reported as Type I (ECA origin); corresponds to Cohen Type II (occipital subtype)	Right ECA-origin Type I PPA at C2–C3 level; bypassed C1 transverse foramen; absence of proximal ipsilateral VA; incidental finding; unruptured left superior hypophyseal artery aneurysm.	Computed tomography angiography (CTA) clarifies bony relationships; it is important for carotid endarterectomy planning and for avoiding posterior circulation compromise during ECA ligation/interventions.
Ozgur et al. [41]	Type I and Type II (angiography series, *n* = 600)	Type I from cervical ICA; Type II from ECA; proximal ipsilateral VA not visualised; both cases had MCA aneurysm.	Documents low incidence (0.33% total) and highlights the need for recognition during catheterisation and intracranial surgery, with attention to coexisting aneurysm.
Ma et al. [34]	Mixed PPA (Type II origin + Type I course; Cohen Type II ‘occipital’ subtype)	PPA arose from proximal ECA (C2) and continued as VA without traversing cervical transverse foramina, consistent with a mixed/occipital Type II course; associated with severe ICA stenosis and bilateral absence of cervical VAs.	Highlights mixed anatomy relevant to carotid stenting and the need to preserve posterior circulation supply when PPA is dominant.
Nariai et al. [38]	Reported as Type I (ECA/occipital origin); corresponds to Cohen Type II (occipital subtype)	Left CCA angiography showed a Type I PPA branching from the ECA (via the OA) at the level of the C2–C3 intervertebral disc and joining the V4 segment; the proximal segment of the left VA was not confirmed, and CTA showed an underdeveloped C3 transverse foramen, supporting VA aplasia with posterior circulation supply via the persistent channel.	Demonstrates contralateral PPA as a retrograde endovascular route for stent-assisted coil embolisation of VA–PICA aneurysm; underscores the need to recognise PPA during access planning.
Ishikawa et al. [26]	Type I (CCA origin) with arch/ICA anomalies	Right Type I PPA arose from right CCA in a patient with aberrant right subclavian artery; proximal right VA aplasia; OA arose from cervical ICA; segmental dysplasia/hypoplasia of ICA segments; associated AcomA aneurysm.	Illustrates that CCA-origin Type I exists and may coexist with complex arch and ICA segmental anomalies; relevant for angiography/endovascular planning and collateral interpretation.
Omura et al. [40]	Type I-like PPA arising from the occipital-system trunk (best interpreted as Cohen Type II ‘occipital’ subtype)	Artery branching from the common trunk of the OA (from ECA) continues as V3, enters the dura via the foramen magnum, and does not pass through the C1 transverse foramen; the ipsilateral VA origin is not visualised; used to reach the distal PICA.	Emergency endovascular treatment (parent artery occlusion) of a ruptured distal PICA aneurysm was performed via the persistent channel, demonstrating that recognising the variant can inform a feasible access strategy.
Ravikanth and Kamalasekar [47]	Type I PPA (ICA origin) with aneurysm and basilar aplasia	Type I PPA arising from the ICA and supplying the posterior circulation in the setting of hypoplastic VAs and an aplastic basilar artery; associated with an anterior communicating artery aneurysm.	Highlights the procedural haemodynamic implications of aneurysm surgery when the posterior circulation supply is carotid-dependent via a persistent proatlantal channel.
Uchino and Tokushige [60]	Type II PPA with normally developed ipsilateral VA	A small left Type II PPA arises from proximal ECA, courses like the OA and fuses with the VA at the level of the foramen magnum; the ipsilateral VA is normally sized; associated with an aberrant right subclavian artery.	Shows that Type II PPA can coexist with a normal ipsilateral VA; relevant for catheterisation and craniovertebral junction surgery where unexpected carotid–vertebral collaterals may be encountered.
Watanabe et al. [64]	Type I PPA with aberrant petrous ICA	Aberrant petrous ICA associated with ipsilateral Type I PPA; contralateral VA and proximal ipsilateral VA absent	Surgical relevance for middle ear surgery and craniovertebral junction procedures; emphasises posterior circulation dependence
Zhao et al. [68]	Type I (ICA-origin; thrombectomy access)	Left Type I proatlantal intersegmental artery from cervical ICA used for acute basilar artery occlusion thrombectomy; right VA tenuous and did not enter the skull, and left VA absent; bilateral PCoAs stunted with poor collateral circulation.	Stent retriever thrombectomy via the Type I channel achieved complete recanalisation (TICI 3) but was followed by brainstem haemorrhage and death; this highlights the need for pre-procedure CTA/DSA to identify access and collateral limitations.
Zuflacht et al. [69]	Type II proatlantal intersegmental artery (persistent first cervical intersegmental artery)	Right common carotid injection demonstrated a large Type II proatlantal intersegmental artery arising from the right ECA, with cross-filling of the contralateral anterior circulation via the anterior communicating artery. In the context of left cervical ICA occlusion and severe atherosclerotic disease/occlusion of the left VA, the basilar artery was supplied exclusively via the right persistent channel.	Demonstrates whole-brain perfusion through a single carotid–PPA axis in advanced occlusive disease; highlights risk of posterior circulation compromise with carotid/ECA interventions and value of recognising this collateral pathway on DSA.
Ito et al. [27]	Reported as Type I (ECA origin); corresponds to Cohen Type II (occipital subtype)	Left Type I PPA originated from the ECA at the C2 level and coursed between the C1 arch and occipital bone (without passing through the C1 transverse foramen) to enter the foramen magnum; left VA not visualised from the subclavian/aortic arch; bilateral PCoAs absent; BA occlusion distal to AICA.	Contact aspiration thrombectomy via the PPA achieved recanalisation (TICI 2b) but was complicated by a large haemorrhagic pontine infarction and severe disability; this underscores considering primitive CVA when vertebral origin is not found.
Rahman et al. [46]	Concurrent Type I and Type II	Right Type I from ICA and left Type II from ECA; occipital arteries arose from the anomalous proatlantal vessels bilaterally; proximal VAs were hypoplastic.	Shows bilateral/asymmetric persistence with OA incorporation.
Xiao et al. [67]	MSCTA series (*n* = 11) with origin-based and posterior-circulation supply classification	Incidence 0.01% on MSCTA; origin-based classification: 2/11 Type I and 9/11 Type II; ipsilateral VA agenesis in all cases; aneurysms in 2/11; proposed four-type supply-based system (Types 1–4).	Largest imaging cohort in the uploaded corpus; supports systematic pre-procedural evaluation of posterior circulation supply patterns and associated vascular anomalies.
Boukobza and Laissy [9]	Mixed Type (II origin + I course; Cohen Type II ‘occipital’ subtype)	Large PPA arising from ECA (C1-C2) with Type I-like suboccipital course (no transverse foramina); OA arose from PPA ; absent ipsilateral VA; hypoplastic contralateral VA; azygos ACA; left CCA from brachiocephalic trunk; kinked cervical ICA aneurysm. Boukobza and Laissy (2025) noted that the course is similar to the ‘mixed PPA’ described by Ma et al. (2019).	Illustrates posterior circulation dependence and procedural planning challenges.
Fu et al. [19]	Type II arising from CCA	Type II PPA arising from the common carotid artery; posterior fossa infarcts in the context of cardiac malformations.	Illustrates rare origin variant and potential stroke-pathway implications.
Gantait et al. [20]	Bilateral Type I with distal PPA stenosis (stented)	Bilateral Type I PPA diagnosed on DSA in a patient with vertebrobasilar stroke/TIA; severe (70–75%) stenosis of the distal left PPA with basilar/PCA opacification through it; both VAs occluded (V3/V4 segments reported).	Shows that the persistent channel can itself become a symptomatic atherosclerotic lesion: balloon angioplasty and stenting of the distal PPA achieved good forward flow and clinical stabilisation, and DSA outperformed non-invasive imaging for delineation.
Present case (index vignette; unpublished)	Type II PPA (occipital subtype) giving rise to the OA	Left PPA arose from the posterior aspect of the ECA opposite the C2/C3 intervertebral disc, bypassed the C1 transverse foramen, gave off the OA, and continued as the V3 segment of the VA before entering the skull via the foramen magnum; the subclavian-origin ipsilateral VA was markedly hypoplastic, and the basilar trunk formed with the dominant contralateral VA.	Didactic ‘index case’ illustrating Cohen Type II occipital-subtype anatomy and the limitation of origin-only labels; relevant to planning ECA/CCA interventions and craniovertebral procedures when posterior circulation supply may be carotid-dependent.

## Clinical significance/discussion

In the present index case, the OA arose from an ECA-origin Type II PPA (occipital subtype) that continued as V3 and entered the skull via the foramen magnum, underscoring why ECA manipulation (e.g., embolisation or ligation) and craniovertebral junction procedures require careful assessment of posterior circulation dependence when a PPA is present [8, 43, 58].

Recognition of PPA is critical for surgical and interventional planning. The vessel may be confused with the persistent hypoglossal artery; extracranial formation of the VA, skull entry via the foramen magnum rather than the hypoglossal canal, and a lower cervical origin level (typically C2–C3 vs. C1/C1–C2 for the hypoglossal artery) favour PPA [3, 15, 25]. The PPA can serve as a conduit for endovascular interventions: emergency parent artery occlusion of a ruptured distal PICA aneurysm [40] and mechanical thrombectomy for basilar occlusion [27, 68] have been performed via a PPA when vertebral access was absent, although outcomes of the latter were limited by brainstem haemorrhage in the setting of poor collateralisation. Conversely, the persistent channel itself may become a target for revascularisation when it is the sole posterior fossa conduit [20, 28]. Multislice CTA can depict PPAs non-invasively [2], and recognition is essential before carotid endarterectomy, atlantoaxial fixation, ECA embolisation/ligation, and skull-base procedures, because hybrid and Type II variants can constitute the exclusive supply to the posterior circulation when VAs are hypoplastic or absent [9, 12, 16, 67, 69] (Table 5).

A key differential diagnosis for a transoccipital/transosseous V3 VA course, reported by Rusu, is a PPA and, less commonly, a PPHA. In contrast to PPAs, which are persistent carotid–vertebrobasilar anastomoses arising from the ICA/ECA (or rarely CCA) and characteristically enter the skull via the foramen magnum with a suboccipital, V3-like sweep, Rusu’s variant represents the native VA (after traversing the C1 transverse foramen) that penetrates the occipital bone through a parasigmoid canal and enters the posterior fossa posteroinferior to the hypoglossal canal, i.e., not through the foramen magnum [13, 45, 49]. The PPHA is discriminated by its more vertical course and trans-hypoglossal canal entry, whereas PPAs retain the foramen magnum entry and a carotid-system origin [13]. Therefore, when faced with an apparent “PPA-like” posterior fossa inflow but the vessel is demonstrably the VA itself with transosseous occipital routing, it is best classified as an unusual V3/intracranial entry variant rather than a PPA [49].

PPA is associated with intracranial and extracranial aneurysms (2/11 in the largest MSCTA cohort [67]), cerebral infarction, and other vascular anomalies including azygos A2, aberrant right subclavian artery, and aortic arch variants [50, 60, 67]. Aneurysms have been reported at the anterior communicating artery [47, 57], middle cerebral artery [41], persistent trigeminal artery [55], subclavian artery [10], and cervical ICA [9]. Haemorrhagic presentations include ruptured aneurysm [29], dural arteriovenous fistula [11], and subarachnoid haemorrhage [37]; ischaemic presentations include posterior fossa infarcts in the setting of rare CCA-origin Type II PPA [19] and vertebrobasilar TIA with distal PPA stenosis amenable to stenting [20]. The most common presenting symptom is dizziness, likely related to underdevelopment of the vertebral and basilar arteries [67]; objective pulsatile tinnitus has also been reported as a presenting feature of Type II PPA [31]. Several Type I configurations underscore posterior circulation vulnerability: bilateral VA absence [7, 22, 36, 57], fetal-type PCA with absent contralateral PCoA [36], and aberrant petrous ICA with contralateral VA absence [64]. CCA-origin Type I with aberrant right subclavian artery has also been documented, with the OA arising from the cervical ICA, supporting a developmental link between the occipital system and proatlantal channels [26]. These associations reinforce the recommendation to survey for coexisting intracranial pathology and arch anomalies when a PPA is identified (Table 5).

In a recent contemporary case-based review, Baskaran et al. (2025) emphasised that Type II PPA is typically an incidental imaging finding, but remains clinically relevant because ECA interventions (e.g., embolisation or ligation), carotid endarterectomy, and cervical spine/skull-base approaches may endanger posterior circulation flow when the proatlantal channel contributes materially to vertebrobasilar perfusion [8]. This broader emphasis aligns with anatomical reviews of persistent fetal carotid-vertebrobasilar anastomoses, which underscore the need to recognise these channels during angiography and skull base/neck surgery [43, 58]. This need is reinforced by microsurgical anatomical work on the vertebrobasilar trunk, which emphasises that detailed recognition of posterior circulation variants underpins safe posterior fossa and skull base interventions [48].

## Conclusions

The persistent proatlantal artery is the most caudal persistent carotid–vertebrobasilar anastomosis and remains underrecognised owing to inconsistent application of the Type I/Type II dichotomy. Origin alone – ICA versus ECA – is insufficient for classification; the cervical course, specifically whether the channel traverses the C1 transverse foramen or follows a suboccipital sweep to the foramen magnum, is the more reliable discriminator. Cohen’s subdivision of Type II into “spinal” (transverse foramen–traversing) and “occipital” (transverse foramen–bypassing) subtypes resolves much of the nomenclature overlap, particularly for ECA/occipital-system channels that mimic a Type I course and have been variably labelled in the literature. Standardised, course-based reporting that explicitly documents the artery of origin, relationship to the C1 transverse foramen, occipital artery incorporation, and ipsilateral VA status will reduce diagnostic ambiguity. Because the PPA frequently serves as the dominant or sole posterior fossa supply, its recognition is essential before carotid endarterectomy, ECA embolisation or ligation, and craniovertebral junction or skull-base surgery. The evidence base remains limited to case reports and small retrospective series; prospective, registry-level data are needed to refine prevalence estimates and to evaluate whether supply-based classifications improve risk stratification and procedural planning.

## Data Availability

The datasets used and analyzed during the current study are available from the corresponding author upon reasonable request.

## Abbreviations

A1: Precommunicating segment of anterior cerebral artery
A2: Postcommunicating segment of anterior cerebral artery
ACom: Anterior communicating artery
ACA: Anterior cerebral artery
AICA: Anterior inferior cerebellar artery
AP: Anteroposterior
AVM: Arteriovenous malformation
BA: Basilar artery
CCA: Common carotid artery
CEA: Carotid endarterectomy
CTA: Computed tomography angiography
DSA: Digital subtraction angiography
ECA: External carotid artery
FM: Foramen magnum
ICA: Internal carotid artery
MCA: Middle cerebral artery
MR: Magnetic resonance
MSCTA: Multislice computed tomography angiography
OA: Occipital artery
PCA: Posterior cerebral artery
PCoA: Posterior communicating artery
PHA: Persistent hypoglossal artery
PICA: Posterior inferior cerebellar artery
PPA: Persistent proatlantal artery
PPHA: Persistent hypoglossal artery (alternative abbreviation)
PTA: Persistent (primitive) trigeminal artery
PTAV: Persistent trigeminal artery variant
SANRA: Scale for the assessment of narrative review articles
SCA: Superior cerebellar artery
TIA: Transient ischaemic attack
TICI: Thrombolysis in cerebral infarction (reperfusion grade)
TP C1: Transverse process of the atlas (C1)
VA: Vertebral artery
V1: V4–vertebral artery segments 1–4
